# Comparison between the Antioxidant and Antidiabetic Activity of Fenugreek and Buckthorn in Streptozotocin-Induced Diabetic Male Rats

**DOI:** 10.1155/2021/7202447

**Published:** 2021-08-27

**Authors:** Mohammed A. Alsieni, Haddad A. El Rabey, Abdulbasit I. Al-Sieni, Madeha N. Al-Seeni

**Affiliations:** ^1^Pharmacology Department, Faculty of Medicine, King Abdulaziz University, Jeddah, Saudi Arabia; ^2^Biochemistry Department, Faculty of Science, University of Tabuk, Tabuk, Saudi Arabia; ^3^Bioinformatics Department, Genetic Engineering and Biotechnology Research Institute, University of Sadat City, P.O. Box 79, Sadat City, Egypt; ^4^Biochemistry Department, Faculty of Science, King Abdulaziz University, Jeddah, Saudi Arabia

## Abstract

This study is aimed at comparing the antidiabetic and antioxidant potential of fenugreek and buckthorn which are commonly used in modulating diabetes in the Middle East. In this study, the antioxidant and antidiabetic activity of the aqueous extracts of the leaf and seed of fenugreek and buckthorn was tested in streptozotocin-induced diabetic male rats fed with a fat-rich diet for 8 weeks. Thirty-six male albino rats were divided into 6 groups (*n* = 6); the 1st group was the negative control. Diabetes was induced in the other 30 rats using streptozotocin, which were then divided into 5 groups; the 2nd was the untreated positive diabetic group, the 3rd was treated with fenugreek leaf aqueous extract, the 4th was treated with the fenugreek seed aqueous extract, the 5th was treated with buckthorn leaf aqueous extract, and the 6th was treated with buckthorn seed aqueous extract. The positive control group showed an increase in blood sugar, glycated hemoglobin, liver function enzymes, lactate dehydrogenase, kidney indices, total cholesterol, triglycerides, low- and very-low-density lipoprotein, immunoglobulins, and lipid peroxidation and a decrease in high-density lipoprotein, albumin, and antioxidant activity. The histology of the liver and testes showed severe histopathological alterations. Rats of groups 4-6 that were treated with the aqueous extract of the leaf and seed extract of fenugreek and buckthorn showed improvement of all biochemical and histopathological parameters. The seed extract of fenugreek and buckthorn showed more antioxidant activity than their leaves.

## 1. Introduction

Diabetes mellitus (DM) is described as chronic hyperglycemia because of a deficiency in either insulin secretion (type 1 DM) or insulin activity (type 2 DM) or both [1, 2]. Type 1 DM is principally occurring because of the obliteration of the insulin-producing pancreatic beta cells in the Langerhans islets because of an autoimmune disease that causes a flat-out insufficiency of insulin [3].

Type 2 DM is the most widely recognized type of diabetes where hyperglycemia occurs because of insulin resistance because of the diminishing function of the target tissue to react appropriately to insulin and dysfunctional *β* cells [4]. In obese pregnant women, gestational diabetes mellitus (GDM) occurs as glucose intolerance in about 7% of all pregnancies, occurring in about >200,000 cases each year [5]; out of these cases, there is a 30%-50% possibility for type 2 DM to occur [6].

Fenugreek (*Trigonella foenum-graecum*, family *Fabaceae*) is developed worldwide for use as a significant fixer in dishes and as a dietary supplement [7]. It has hypolipidemic and antioxidant action [8]. It was likewise utilized for balancing diabetes in many nonindustrial nations because of its content of triacylglycerol and phosphatidylethanolamine, which are the major molecular species recognized in the neutral and polar lipid parts of fenugreek seeds, respectively [9, 10].

It was additionally discovered that fenugreek improves diabetic hypertensive nephropathy by suppression of oxidative stress in the kidney and decreases renal cell apoptosis and fibrosis in streptozotocin-induced neonatal diabetic rats [11, 12].

The antioxidant action of fenugreek methanolic extract shows scavenging of hydroxyl radicals (^·^OH) and inhibition of hydrogen peroxide-induced lipid peroxidation in rat liver mitochondria [13]. Furthermore, Kumar et al. [9] detailed that the hydroalcoholic extract of fenugreek ameliorates different disabilities related to physical fatigue in rats exposed to the obesity stacked constrained swim test.

*Zizyphus spina-christi* (buckthorn) of the family *Rhamnaceae* is regularly utilized in conventional medicine in treating obesity, liver complaints, fever, urinary troubles, diabetes, diarrhea, stomach-related disorders, skin diseases, weakness, and sleep disorder [14, 15]. In addition, the cleansed peptides from *Z. jujuba* proteins forestall oxidative responses and can be utilized for medicinal purposes and food conservation [16].

The pharmacological antidiabetic activity of buckthorn is attributed to controlling meal-derived glucose retention [15, 17, 18]. Buckthorn likewise ameliorates schistosomiasis liver granuloma, fibrosis, and oxidative stress through downregulation of fibrinogenic motioning in mice [19]. Besides, it has antioxidant and anti-inflammatory properties [20–24]. Likewise, extracts from the fruits and seeds of *Z. spina-christi* displayed an antimicrobial action against *Bacillus subtilis*, *E*. *coli*, and *Streptococcus pyogenes* [25]. The antioxidant and antidiabetic activity of the aqueous extract of the leaf and seed of fenugreek and buckthorn was assessed in streptozotocin- (STZ-) induced diabetic male rats under hypercholesterolemic conditions.

## 2. Materials and Methods

### 2.1. Test Materials and Diet

The leaves and seeds of fenugreek were purchased from an agricultural shop at Jedda, KSA, and the buckthorn leaves and seeds were also collected from buckthorn trees at Jedda. All plant materials were defined by a botanist, and herbal specimens were deposited at the Herbarium of King Abdulaziz University.

During this current study, rats ate *ad libitum* the fat-rich diet as stated by El Rabey et al. [14].

### 2.2. Fenugreek and Buckthorn Seed Aqueous Extract Preparation

The aqueous extracts were prepared as indicated by the method of Sharma et al. [26]. The dry leaves and seeds of buckthorn and fenugreek were washed with refined water, sun-dried for 72 h, and processed in a blender. 500 g of the powder was soaked in 5-liter refined water for 72 h under constant shaking with intervals of 30 min. The blend was filtered using 250 mm filter paper, and afterward, the filtrate was freeze-dried at -52°C. A 200 g semisolid product was created. To acquire the suggested concentration, the appropriate obesity of the semisolid product was disintegrated in the reasonable measure of refined water.

### 2.3. Experimental Animals and Experiment Design

36 Sprague-Dawley male rats of East Asian origin weighing from 180 ± 15 to 200 ± 18 g (two months of age) were purchased from King Fahad Center for Medical Research, King Abdulaziz University, KSA. All lab tests were conducted at King Fahad Center for Medical Research under a convention affirmed by the Bioethical Board of Trustees of King Abdulaziz University.

The rats were distributed in 6 stainless cages (*n* = 6) and held fourteen days under perception for acclimatization earlier before the beginning of the trial. During this investigation, the test diet and water were accessible *ad libitum*. The trial room condition was changed at 24°C ± 1°C and 55% ± 5% humidity, with a 12 h light-dim cycle.

After the acclimatization period, the rats were filtered into 6 groups (*n* = 6): the first group was the negative control group (G1) that received a solitary tail vein injection of 0.1 mol/l citrate buffer (pH 4.5), and the other 30 rats were intraperitoneally injected with freshly arranged STZ (65 mg/kg b.w.) in a 0.1 mol/l citrate buffer, in the wake of fasting for 12 h as in Qusti et al. [27].

Rats with fasting blood glucose higher than 200 mg/dl following five days of injection are viewed as diabetic.

These diabetic rats were randomly divided into five groups as follows: the second group was the positive control group that was left with no treatment, the third group (G3) was treated with 200 mg/kg b.w. of fenugreek leaf aqueous extract, the fourth group (G4) was treated with 200 mg/kg b.w. of fenugreek seed aqueous extract, the fifth group (G5) was treated with 200 mg/kg b.w. buckthorn leaf aqueous extract, and the sixth group was treated with 200 mg/kg b.w. buckthorn seed aqueous extract. The treatment portion was conveyed by a stomach gavage tube, and the experiment was continued for about two months.

### 2.4. Blood Collection

After the two-month trial period, rats were fasted for 12 h and afterward euthanized by cervical dislocation. Blood tests were gathered from the heart for serum readiness.

All rats were analyzed; one kidney was washed by saline buffer and saved cold in ice for kidney tissue homogenate preparation. A piece of the liver and one testis were washed in saline buffer and afterward fixed in 10% formalin for histopathological preparation.

### 2.5. Kidney Tissue Homogenate

The kidney tissue homogenate was prepared according to the method described by Al-Seeni et al. [28].

### 2.6. Biochemical Analyses

The blood glucose level was colorimetrically assessed using a Human Kit (Germany) as indicated by Barham and Trinder [29]. In addition, serum hemoglobin A1c was assessed by the guidance of the supplier of the Glycohemoglobin Reagent Kit (Pointe Scientific Inc., USA).

Lactate dehydrogenase (LDH) action was assessed spectrophotometrically in serum as indicated by Martinek [30] using the Teco Diagnostics Kit (USA).

Serum triglycerides (TG) and total cholesterol (TC) were assessed using the method of Young [31], and serum high-density lipoproteins (HDL) were assessed using the technique of Naito [32], while serum low-density lipoproteins (LDL) and the very-low-density lipoproteins (VLDL) were determined by the following equation of Srivastava et al. [33]:

(1) $$\begin{array}{c} \text{LDL}\;\;\left(\text{mg}/\text{dl}\right)=\text{total}\;\;\text{cholesterol}–\text{HDL}–\left(\frac{\text{triglycerides}}{5}\right),\\ \text{VLDL}=\frac{\text{triglycerides}}{5}. \end{array}$$

Human (Germany) packs were used for assessing liver function in the serum. Alanine aminotransferase (ALT), gamma-glutamyl transferase (*γ*-GT), and alkaline phosphatase (ALP) were used as indicated by the guidelines of the suppliers using the strategies of Thefeld et al. [34], Thomas [35], and Schlebusch et al. [36]. Serum albumin was assessed in serum using the Sigma-Aldrich pack (United States) following the method of Doumas et al. [37].

Urea was colorimetrically assessed in serum following the method of Fawcett and Scott [38] using the Human Kit (Germany). Serum creatinine (CRE) and uric acid were likewise assessed using the Human Kit (Germany) following the method of Bartels et al. [39]. All tests were done with the guidance of the supplier.

Serum immunoglobulins (IgA, IgM, and IgG) were assessed using the directions of the GenWay Biotech Kit (USA).

The antioxidant enzymes, namely, catalase (CAT), superoxide dismutase (SOD), and glutathione-S-transferase (GST), were assessed in the kidney tissue homogenate using the Biodiagnostic unit (Egypt), following the directions of the suppliers and the techniques of Aebi [40], Nishikimi et al. [41], and Dolphin et al. [42]. In addition, malondialdehyde (MDA) levels were assessed in the kidney tissue homogenate using the Biodiagnostic unit (Egypt), following the method of Ohkawa et al. [43].

### 2.7. Histopathology

Briefly, tissues of the liver and testes were washed in saline after dissection, fixed in 10% formalin, processed in an ascending series of alcohol routinely, and then embedded in paraffin. After that, 5 *μ*m segments of liver and testis were prepared, stained in hematoxylin and eosin (H&E) dye as indicated by the method of Drury et al. [44], and afterward inspected and captured under an Olympus light microscope with a digital camera.

### 2.8. Statistical Analysis

Data were analyzed using the SPSS (Statistical Program for Sociology Scientists) program version 23.0 (IBM® SPSS® Statistics, Armonk, N.Y., USA). The standard errors (SE), trial of importance (*t*-test), and one-way analysis of variance (ANOVA) were determined. Charts were plotted to utilize SigmaPlot programming (AutoSignal V1.7).

## 3. Results

### 3.1. Antioxidants and Lipid Peroxidation

**Figure 1:**
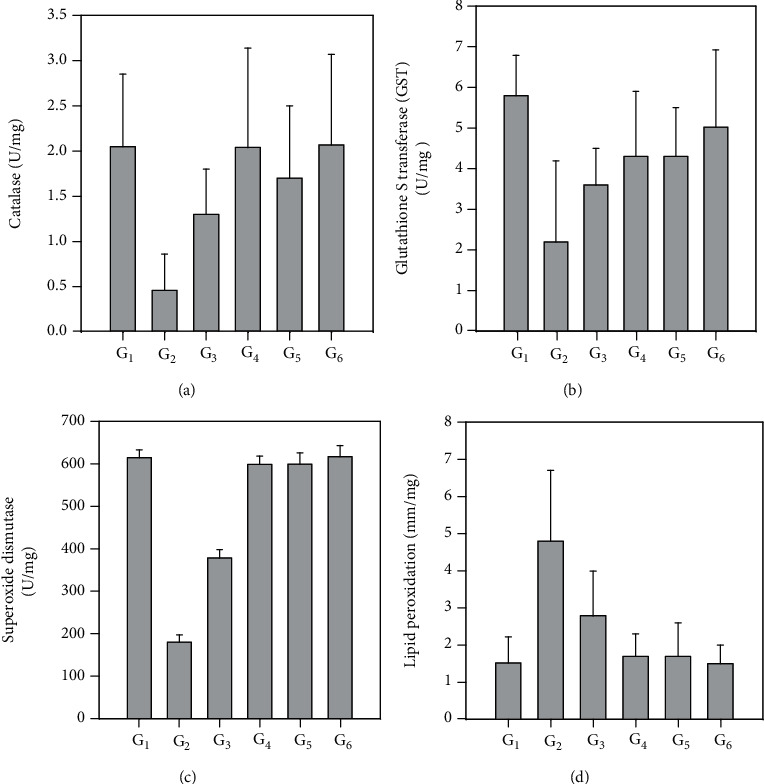
Effect of treating STZ-induced diabetes with fenugreek and buckthorn aqueous extracts on antioxidant and lipid peroxidation in kidney tissue homogenate of the rats under study: (a) CAT, (b) GST, (c) SOD, and (d) lipid peroxidation. G1: negative control; G2: positive diabetic group; G3: diabetic group treated with fenugreek leaf; G4: diabetic group treated with fenugreek seed; G5: diabetic group treated with buckthorn leaf; G6: diabetic group treated with buckthorn seed.

Figures 1(a)–1(d) and Table 1 (sup.) show the effect of treating STZ-diabetic rats with fenugreek and buckthorn aqueous extracts of leaves and seeds on antioxidant enzymes and lipid peroxidation. The examined antioxidant enzymes were significantly (*P* < 0.001) decreased due to induction of diabetes in G2 rats compared with the negative control (G1).

Treating the diabetic rats in G3, G4, G5, and G6 with fenugreek and buckthorn aqueous extracts of leaves and seeds significantly (*P* < 0.001) increased the antioxidant enzymes compared with the positive control (G2). Interestingly, lipid peroxidation was significantly (*P* < 0.001) increased in the positive control group because of diabetes induction compared with G1 and decreased by treating with fenugreek and buckthorn aqueous extracts of leaves and seeds in G3, G4, G5, and G6 compared with the positive control (G2).

### 3.2. Serum Glucose and Glycated Hemoglobin A1c

**Figure 2:**
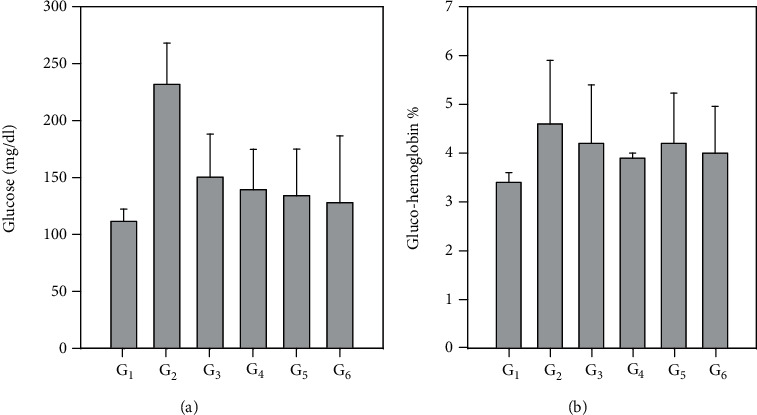
Effect of treating STZ-induced diabetes with fenugreek and buckthorn aqueous extracts on glucose levels (a) and glycated hemoglobin (b) of the rats under study. G1: negative control; G2: positive diabetic group; G3: diabetic group treated with fenugreek leaf; G4: diabetic group treated with fenugreek seed; G5: diabetic group treated with buckthorn leaf; G6: diabetic group treated with buckthorn seed.

Figure 2 and Table 2 (sup.) show the effect of treating STZ-diabetic rats with fenugreek and buckthorn aqueous extracts of leaves and seeds on serum glucose and glucohemoglobin (glycated hemoglobin). Induction of diabetes in the positive control group (G2) significantly raised glucose and the glucohemoglobin compared with the negative control, while treating with fenugreek and buckthorn aqueous extracts of leaves and seeds in G3-G6 significantly decreased these parameters moving toward the normal values.

### 3.3. Lipid Profile

Table 1 shows the effect of treating STZ-diabetic rats with fenugreek and buckthorn aqueous extracts of leaves and seeds on lipid profile. The total cholesterol, triglycerides, LDL, and VLDL were fundamentally increased in the positive control group compared with the negative control, while HDL was significantly decreased. By treating the diabetic rats in G3, G4, G5, and G6 with the aqueous extracts of leaves and seeds of fenugreek and buckthorn, the total cholesterol, triglycerides, LDL, and VLDL significantly decreased (*P* < 0.001) and the levels of HDL increased.

### 3.4. Liver Function

**Figure 3:**
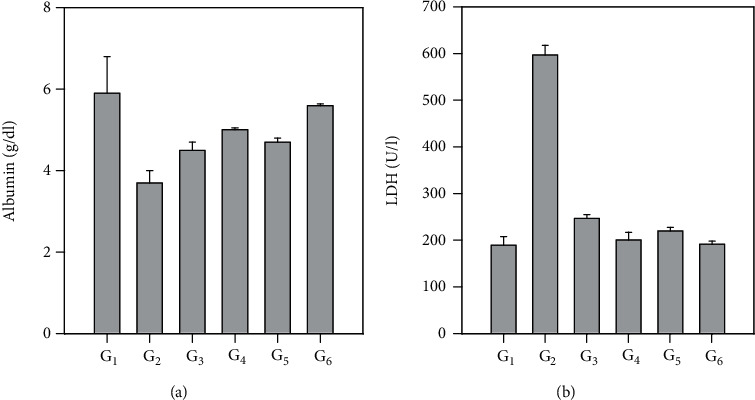
Effect of treating STZ-induced diabetes with fenugreek and buckthorn aqueous extracts on serum albumin (a) and lactate dehydrogenase (b) of the rats under study. G1: negative control; G2: positive diabetic group; G3: diabetic group treated with fenugreek leaf; G4: diabetic group treated with fenugreek seed; G5: diabetic group treated with buckthorn leaf; G6: diabetic group treated with buckthorn seed.

Table 2, Table 2 (sup.), and Figure 3 show the effect of treating the STZ-diabetic rats with the aqueous extracts of the leaves and seeds of fenugreek and buckthorn on liver function parameters: ALT, *γ*-GT, and ALP. Induction of diabetes in G2 significantly increased the levels of the examined liver function enzymes (ALT, *γ*-GT, and ALP) compared with the negative control, although treating the diabetic rats in G3, G4, G5, and G6 with the aqueous extracts of leaves and seeds of fenugreek and buckthorn significantly (*P* < 0.001) decreased the elevated liver function enzyme action.

In addition, Figure 3 shows that serum albumin was decreased by diabetes induction in G2, although lactate dehydrogenase was increased compared with the negative control group. Treating the diabetic rats in groups 3, 4, 5, and 6 with the aqueous extracts of the leaves and seeds of fenugreek and buckthorn restored these modified values of albumin and lactate dehydrogenase to their normal levels.

### 3.5. Kidney Function Parameters

Table 3 shows the effect of treating the STZ-diabetic rats with the aqueous extracts of the leaves and seeds of fenugreek and buckthorn on kidney function (CRE, urea, and uric acid). Rats of the positive control group showed elevated kidney function compared with the negative control. The difference was highly significant (*P* < 0.001), while treating these diabetic rats in G3-G6 with the aqueous extracts of the leaves and seeds of fenugreek and buckthorn significantly (*P* < 0.001) enhanced restoring these kidney function parameters almost to the typical levels.

### 3.6. Immunoglobulins

Table 4 shows the effect of treating the STZ-diabetic rats with the aqueous extracts of the leaves and seeds of fenugreek and buckthorn on immunoglobulins (IgA, IgM, and IgG). Induction of diabetes in the positive control group (G2) significantly raised IgA, IgM, and IgG compared with the negative control while treating these diabetic rats in G3-G6 with the aqueous extracts of the leaves and seeds of fenugreek and buckthorn significantly (*P* < 0.001) decreased these parameters moving toward the normal values.

Data are represented as the mean ± SE. *t*-test values: ∗∗∗ means significant at *P* < 0.001. ANOVA analysis: within each row, means with different superscripts (a, b, c, d, e, or f) are significantly different at *P* < 0.05, whereas means with superscripts that have the same letters mean that there is no significant difference at *P* < 0.05. LSD: least significant difference.

### 3.7. Histopathology

#### 3.7.1. Liver

**Figure 4:**
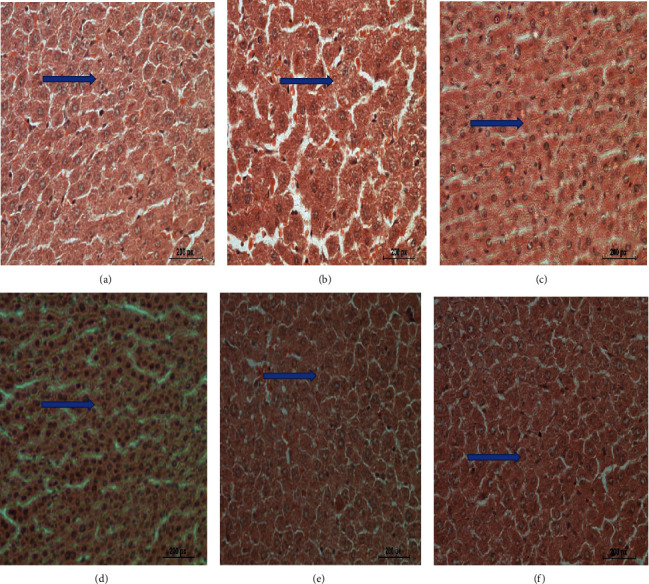
Effect of treating STZ-induced diabetes with fenugreek and buckthorn aqueous extracts on liver tissues. (a) Negative control with normal hepatic tissues (arrow). (b) Positive diabetic group with distorted hepatocytes and loss of normal architecture (arrow). (c) Diabetic group treated with fenugreek leaf with nearly normal hepatic tissues (arrow). (d) Diabetic group treated with fenugreek seed with nearly normal hepatic tissues (arrow). (e) Diabetic group treated with buckthorn leaf with nearly normal hepatic tissues (arrow). (f) Diabetic group treated with buckthorn seed with normal hepatic tissues (arrow) (H&E ×400).

Figure 4(a) shows the normal hepatic tissue of the negative control group.  Figure 4(b) shows mutilated hepatic tissue due to diabetes induction in the positive control group (G2) showing mutilated hepatocytes, striation, vacuolation, and loss of the normal architecture just as increased leucocytic infiltrations in the middle of vacuolations.

Figure 4(c) shows the slightly changed hepatic tissue of the diabetic group treated with the aqueous extract of fenugreek leaf.  Figure 4(d) shows the almost typical hepatic tissue of the diabetic group treated with the aqueous extract of fenugreek seed.  Figure 4(e) shows the slightly altered hepatic tissue of the diabetic group treated with the aqueous extract of a buckthorn leaf.  Figure 4(f) shows the almost typical hepatic tissue of the diabetic group treated with the aqueous extract of buckthorn seed.

#### 3.7.2. Testis

**Figure 5:**
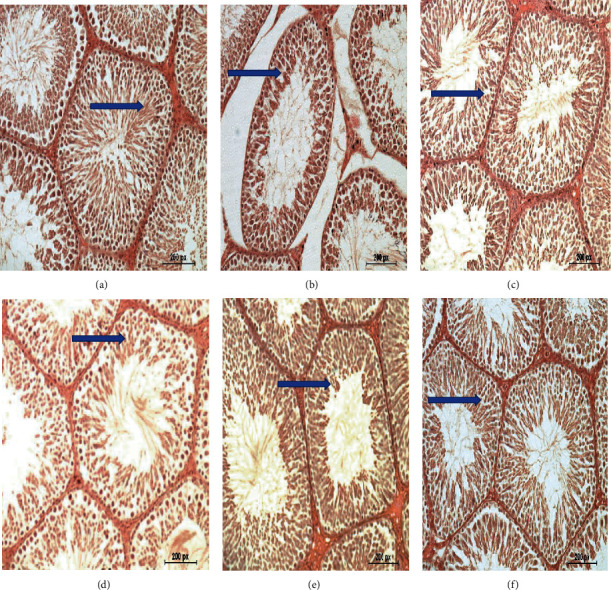
Effect of treating STZ-induced diabetes with fenugreek and buckthorn aqueous extracts on testis tissues. (a) Negative control with normal testicular tissues (arrow). (b) Positive diabetic group with degeneration of the seminiferous tubules and spermatocytes (arrow). (c) Diabetic group treated with fenugreek leaf with nearly normal testicular tissues (arrow). (d) Diabetic group treated with fenugreek seed with nearly normal testicular tissues (arrow). (e) Diabetic group treated with buckthorn leaf with nearly normal testicular tissues (arrow). (f) Diabetic group treated with buckthorn seed with normal testicular tissues (arrow) (H&E ×400).

Figure 5(a) shows the typical testicular tissue of the negative control group.  Figure 5(b) shows mutilated testicular tissue due to diabetes induction in the positive control group (G2) with degeneration of the seminiferous tubules, lysis of spermatocytes, and necrosis of the spermatids.  Figure 5(c) shows the slightly changed testicular tissue of the diabetic group treated with the aqueous extract of a fenugreek leaf.  Figure 5(d) shows the almost normal testicular tissue of the diabetic group treated with the aqueous extract of fenugreek seed.  Figure 5(e) shows the slightly altered testicular tissue of the diabetic group treated with the aqueous extract of buckthorn leaf.  Figure 5(f) shows the almost typical testicular tissue of the diabetic group treated with the aqueous extract of buckthorn seed.

## 4. Discussion

Numerous patients lean toward treatments using herbs and depend on traditional medicine to keep away from the unsafe side effects brought about by using synthetic medicines. Medicinal plants contain an assortment of natural products that show considerable antioxidant, antidiabetic, anticancer, and anti-hypercholesterolemic activity [16, 27, 45, 46].

In this investigation, we study the antioxidant and antidiabetic action of the aqueous extract of the leaf and seed of fenugreek and buckthorn in STZ-induced diabetic male rats. Diabetes induction using STZ increased blood glucose, glycated hemoglobin A1c, liver and kidney function parameters, immunoglobulins, lipid peroxidation, and reduction of the antioxidant enzymes compared with the control group. Induction of diabetes in the positive control group increased both glucose and glycated hemoglobin because of the injury of *β*-pancreatic cells that secrete insulin causing a decrease in insulin delivery and lessening of glucose admission by the cells that cause an increment of glucose expanding the oxidative stress and influencing other fundamental capacities [2, 27]. The increment in lipid peroxidation and the diminishing of the antioxidant enzymes compared with the control group reflected the higher oxidative stress which came about because of STZ-induced diabetes [47, 48].

In addition, liver function enzymes were likewise increased and albumin was decreased as an aftereffect of STZ induction in the positive control group. This outcome is comparable with those of Al-Malki and El Rabey [47] and Qusti et al. [27]. In addition, albumin levels were decreased by diabetes induction and treatment with the aqueous extract of fenugreek and buckthorn.

In the positive control group, the height of lactate dehydrogenase levels in the blood because of diabetes induction is attributed to cell harm [27]. Treating diabetic animals with the aqueous extract of fenugreek and buckthorn restored LDH to its normal levels. The increase in immunoglobulins (IgA, IgM, and IgG) in the positive control is consistent with Al-Malki and El Rabey [47] and Attia et al. [48]. The increase in these markers reflects the stressed immune system state resulting from diabetes induction using STZ.

Essentially, the induction of diabetes likewise increased the absolute cholesterol, triglycerides, and low-density lipoprotein and decreased the high-density lipoprotein because of the oxidative stress which came about from the induction of diabetes [27, 48]. The rise of the kidney parameter lists because of STZ-induced diabetes is reliable with the significant entanglements of diabetes and with past investigations [27, 48, 49].

All these altered parameters were restored almost to their normal levels in the groups treated with the aqueous extracts of both fenugreek and buckthorn leaves and seeds because of their antioxidant and antidiabetic action. The enhancing effect of fenugreek might be because its substance has the major subatomic species, for example, triacylglycerol and phosphatidylethanolamine, and different steroids and alkaloids, for example, trigonelline, trigocoumarin, and trimecoumarine [2, 50, 51].

In mice, Choudhary et al. [52] stated that dietary organization of 1 and 2% fenugreek seeds brought about an increment of decreased glutathione (GSH) and GST activity in the liver homogenate of rats and has no considerable change in SOD and catalase (CAT) [52]. Fenugreek likewise showed anti-inflammatory and antioxidant activity and decreased lipid peroxide by applying valuable effects on oxidative stress that came about because of diabetes mellitus [53]. In addition, Gupta et al. [54] and Broca et al. [55] announced that the antidiabetic effect of fenugreek is because of the decrease of the glucose assimilation rate and the deferral of gastric emptying. Along these lines, the ascent in glucose levels is forestalled following consumption of 4-hydroxyisoleucine amino that likewise invigorates insulin emission and expansion in the quantity of insulin receptor locales to consume cell glucose and forestalling the quick ingestion of glucose in the small digestive tract that causes glucose maintenance, separately.

In a similar study, the curative effect of buckthorn seed and leaf is upheld by other past examinations [14, 22–24]. The leaf extract of buckthorn showed efficacy against diabetic action and forestalled liver fibrosis and oxidative stress through downregulation of fibrinogenic flagging [17, 19]. The seeds of buckthorn have antidiabetic action in rats [15] by enhancing the overall values of diabetes, carb digestion enzymes, and lipid profile.

In the positive control group, induction of diabetes altered the histology of the liver and testis of the control positive group [27, 47], although by treating the diabetic groups with the aqueous extract of fenugreek and buckthorn, these tissues restored almost typical appearance. The histopathological harm of the considered organs in STZ-induced diabetic rats is steady with Ahmed, Ouvrier et al. [56], and Al-Malki and El Rabey [47]. This recuperating effect is ascribed to the antioxidant and antidiabetic properties of the phenol enzymes comprising fenugreek and buckthorn close to their substance of other antioxidant and antidiabetic characteristic products.

Induction of diabetes by STZ destroyed the beta-insulin-emitting cells of the pancreas, raised the oxidative stress that leads to the rise of kidney and liver capacities, increased the lipid peroxidation and lipid profile, decreased antioxidant enzymes, and caused a change of the histology of liver and testis of the control positive group. The aqueous extracts of fenugreek and buckthorn leaf and seed improved biochemical and histological parameters in STZ-induced diabetes in male rats. These curative effects are ascribed to their active constituents of the antioxidant and antidiabetic materials. The seed extract of fenugreek and buckthorn showed more antioxidant and antidiabetic effects than their leaves.

## Figures and Tables

**Table 1:** Effect of treating STZ-induced diabetes with fenugreek and buckthorn aqueous extract on the lipid profile in male rats.

Statistics and tests		G1 (-ve control)	G2 (+ve control)	G3 Fenugreek leaf	G4 Fenugreek seed	G5 Buckthorn leaf	G6 Buckthorn seed
Total cholesterol (mg/dl)	Mean ± SD						
	LSD 0.05 = 16.78	155.9 ± 5.9^a^	268.1 ± 10.3^b^	181.1 ± 19.4^c^	155.2 ± 8.3^d^	173.9 ± 18.3^e^	160.4 ± 10.5^f^
	*t*-test	—	2.509^∗∗∗^	3.042^∗∗∗^	0.184^∗∗∗^	2.285^∗∗∗^	2.938^∗∗∗^

Triglyceride (mg/dl)	Mean ± SD						
	LSD 0.05 = 10.26	100.8 ± 21.4^a^	184.2 ± 10.8^b^	149.03 ± 18.05^c^	123.1 ± 23^d^	124.4 ± 40.3^e^	109.4 ± 5.7^f^
	*t*-test	—	3.411^∗∗∗^	0.157^∗∗∗^	1.735^∗∗∗^	1.419^∗∗∗^	4.586^∗∗∗^

HDL (mg/dl)	Mean ± SD	58.8 ± 21.4^a^	34.2 ± 10.8^b^	44.03 ± 18.05^c^	50.1 ± 2.3^d^	47.4 ± 4.3^e^	59.4 ± 5.7^f^
	LSD 0.05 = 7.16						
	*t*-test	—	3.411^∗∗∗^	−0.157^∗∗∗^	−1.735^∗∗∗^	−1.419^∗∗∗^	−4.586^∗∗∗^

LDL (mg/dl)	Mean ± SD						
	LSD 0.05 = 8.08	80.8 ± 2.4^a^	181.2 ± 10.8^b^	94.03 ± 8.05^c^	73.1 ± 2.3^d^	99.4 ± 4.3^e^	79.4 ± 5.7^f^
	*t*-test	—	3.411^∗∗∗^	0.157^∗∗∗^	1.735^∗∗∗^	1.419^∗∗∗^	4.586^∗∗∗^

VLDL (mg/dl)	Mean ± SD	20.8 ± 2.4^a^	53.2 ± 1.8^b^	36.03 ± 1.8.05^c^	31.1 ± 2.3^f^	24.4 ± 40.3^d^	21.4 ± 5.7^e^
	LSD 0.05 = 2.26						
	*t*-test	—	3.411^∗∗∗^	0.157^∗∗∗^	1.735^∗∗∗^	1.419^∗∗∗^	4.586^∗∗∗^

Data are represented as the mean ± SE. *t*-test values: ∗∗∗ means significant at *P* < 0.001. ANOVA analysis: within each row, means with different superscripts (a, b, c, d, e, or f) are significantly different at *P* < 0.05, whereas means with superscripts that have the same letters mean that there is no significant difference at *P* < 0.05. LSD: least significant difference.

**Table 2:** Effect of treating STZ-induced diabetes with fenugreek and buckthorn aqueous extract on liver function enzymes in male rats under study.

Statistics and tests		G1 (-ve control)	G2 (+ve control)	G3 Fenugreek leaf	G4 Fenugreek seed	G5 Buckthorn leaf	G6 Buckthorn seed
ALT (IU/l)	Mean ± SD						
	LSD 0.05 = 3.79	35.1 ± 5.8^a^	59.6 ± 5.4^d^	44.2 ± 4.8^c^	42.6 ± 2.7^d^	45.3 ± 7.7^e^	34.9 ± 6.7^f^
	*t*-test	—	−7.585^∗∗∗^	2.741^∗∗^	6.701^∗∗^	0.38^∗∗^	1.493^∗∗^

*γ*-GT (IU/l)	Mean ± SD						
	LSD 0.05 = 1.52	32.0 ± 1.04^b^ 9-48	46 ± 1.2^a^	44 ± 1.9^c^	29 ± 0.9^d^	38 ± 1.2^e^	32.1 ± 0.9^f^
	*t*-test	—	−2.129^∗∗∗^	1.407^NS^	0.51	0.076	3.352^∗∗^

Alkaline phosphatase (IU/l)	Mean ± SD						
	LSD 0.05=18.73	151.4 ± 22.7^a^	244.3 ± 16.4^b^	163.7 ± 7.4^c^	153.5 ± 1.2^d^	159.2 ± 11.2^e^	148.7 ± 28.1^f^
	*t*-test	—	−0.617^∗∗∗^	2.849^∗∗∗^	1.391^∗∗∗^	1.182^∗∗^	2.532^∗∗∗^

Data are represented as the mean ± SE. *t*-test values: ∗∗∗ means significant at *P* < 0.001. ANOVA analysis: within each row, means with different superscripts (a, b, c, d, e, or f) are significantly different at *P* < 0.05, whereas means with superscripts that have the same letters mean that there is no significant difference at *P* < 0.05. LSD: least significant difference.

**Table 3:** Effect of treating STZ-induced diabetes with fenugreek and buckthorn aqueous extract on kidney function indices in male rats.

Statistics and tests		G1 (-ve control)	G2 (+ve control)	G3 Fenugreek leaf	G4 Fenugreek seed	G5 Buckthorn leaf	G6 Buckthorn seed
Urea (mg/dl)	Mean ± SD						
	LSD 0.05 = 6.57	24.4 ± 6.9^a^	47.4 ± 3.9^b^	33.1 ± 45.5^c^	29.9 ± 5.5^d^	35.02 ± 7.6^e^	24.2 ± 7.8^f^
	*t*-test	—	0.765^∗∗∗^	0.056^∗∗∗^	3.233^∗∗∗^	2.41^∗∗∗^	5.682^∗∗∗^

CRE (mg/dl)	Mean ± SD						
	LSD 0.05 = 0.34	0.7 ± 0.2^a^	1.2 ± 0.4^b^	1.0 ± 0.2^c^	0.9 ± 0.03^d^	0.9 ± 0.2^e^	0.8 ± 0.4^f^
	*t*-test	—	−2.158^∗∗^	0.747^∗^	2.802^∗^	3.316^∗^	3.092^∗^

Uric acid (mg/dl)	Mean ± SD						
	LSD 0.05 = 0.74	3.5 ± 0.5^a^	8.9 ± 1.2^b^	5.6 ± 0.4^c^	4.6 ± 1.0^f^	4.9 ± 0.9^d^	3.8 ± 0.7^e^
	*t*-test	—	−2.399^∗∗∗^	1.088^∗∗∗^	1.984^∗∗∗^	1.451^∗∗∗^	1.959^∗∗∗^

Data are represented as the mean ± SE. *t*-test values: ∗∗∗ means significant at *P* < 0.001. ANOVA analysis: within each row, means with different superscripts (a, b, c, d, e, or f) are significantly different at *P* < 0.05, whereas means with superscripts that have the same letters mean that there is no significant difference at *P* < 0.05. LSD: least significant difference.

**Table 4:** Effect of treating STZ-induced diabetes with fenugreek and buckthorn aqueous extract on immunoglobulins (IgA, IgM, and IgG) in male rats.

Statistics and tests		G1 (-ve control)	G2 (+ve control)	G3 Fenugreek leaf	G4 Fenugreek seed	G5 Buckthorn leaf	G6 Buckthorn seed
IgA (mg/dl)	Mean ± SD						
	LSD 0.05 = 20.09	104.1 ± 39.2^a^	251.3 ± 9.6^b^	150.6 ± 14.7^c^	112.2 ± 9.4^d^	158.6 ± 4.8^e^	104.7 ± 6.3^f^
	*t*-test	—	3.202^∗∗∗^	3.133^∗^	2.646^∗^	2.663^∗^	3.669^∗^

IgM (mg/dl)	Mean ± SD						
	LSD 0.05 = 18.14	134.2 ± 7.04^b^	244.2 ± 9.7^a^	196.7 ± 6.2^c^	149.5 ± 6.4^d^	150.2 ± 8.2^e^	142.8 ± 6.2^f^
	*t*-test	—	2.955^∗^	13.789^∗∗∗^	4.157^∗^	8.97^∗∗∗^	17.269^∗∗∗^

IgG (mg/dl)	Mean ± SD						
	LSD 0.05 = 42.49	804.8 ± 27.9^a^	1103.7 ± 27.5^b^	922 ± 21.5^c^	859.5 ± 26.98^d^	928.7 ± 23.5^e^	808.2 ± 22.9^f^
	*t*-test	___	1.958	3.132^∗^	5.255^∗∗^	2.687^∗^	1.724

## Data Availability

The data used to support the findings of this study are included within the article and the supplementary files.

## Abbreviations

ALP: Alkaline phosphatase
ALT: Alanine aminotransferase
b.w.: Body weight
CAT: Catalase
CRE: Creatinine
DM: Diabetes mellitus
G1: The 1st group is the negative control that received a single tail vein injection of 0.1 mol/l citrate buffer
G2: The second group is the positive control diabetic group (intraperitoneally injected with freshly prepared STZ (65 mg/kg b.w.) in a 0.1 mol/l citrate buffer)
G3: The 3rd group is a diabetic group as in G2 and was treated with 200 mg/kg b.w. of fenugreek leaf aqueous extract
G4: The 4th group is a diabetic group as in G2 and was treated with 200 mg/kg b.w. of fenugreek seed aqueous extract
G5: The 5th group is a diabetic group as in G2 and was treated with 200 mg/kg b.w. buckthorn leaf aqueous extract
G6: The 6th group is a diabetic group as in G2 and was treated with 200 mg/kg b.w. buckthorn seed aqueous extract
GGT (*γ*-GT): Gamma-glutamyl transferase
GSH: Glutathione
GST: Glutathione-S-transferase
HbA1C: Glycated hemoglobin Alc
HDL: High-density lipoprotein
H&E: Hematoxylin and eosin
IgA: Immunoglobulin A
IgG: Immunoglobulin G
IgM: Immunoglobulin M
LDH: Lactate dehydrogenase
LDL: Low-density lipoprotein
MDA: Malondialdehyde
^·^OH: Hydroxyl radicals
ROS: Reactive oxygen species
SOD: Superoxide dismutase
STZ: Streptozotocin
TC: Total cholesterol
TG: Triglycerides
U/g: Unit per gram
U/l: Unit per liter
U/ml: Unit per milliliter
UA: Uric acid
VLDL: Very-low-density lipoprotein.

## Supplementary Materials

Supplementary 1 Table 1 (sup.): effect of treating streptozotocin-induced diabetes with fenugreek and buckthorn aqueous extract on antioxidants and lipid peroxidation in male rats.

Supplementary 2 Table 2 (sup.): effect of treating streptozotocin-induced diabetes with fenugreek and buckthorn aqueous extract on glucose, glycated hemoglobin, albumin, and lactate dehydrogenase in male rats.
