# Supplementary material for: Comparison between the Antioxidant and Antidiabetic Activity of Fenugreek and Buckthorn in Streptozotocin-Induced Diabetic Male Rats
Source: Biomed Res Int. 2021 Aug 27;2021:7202447. doi: 10.1155/2021/7202447 (PMC8420976; doi:10.1155/2021/7202447)
Supplement: Supplementary 1 — Table 1 (sup.): effect of treating streptozotocin-induced diabetes with fenugreek and buckthorn aqueous extract on antioxidants and lipid peroxidation in male rats. [file 7202447.f1.docx]

Table 1 (sup.): Effect of treating streptozotocin induced diabetes with fenugreek and buckthorn aqueous extract on antioxidants and lipid peroxidation in male rats

| **Statistics and tests** | | **G1**  **(-ve control)** | **G2**  **(+ve control)** | **G3**  **Fenugreek leaf** | **G4**  **Fenugreek seed** | **G5**  **Buckthorn leaf** | **G6**  **Buckthorn seed** |
| --- | --- | --- | --- | --- | --- | --- | --- |
| **Catalase**  **(U/mg)** | Mean ± SD | 2.05±0.8^a^ | 0.46± 0.4^b^ | 1.3± 00.5^c^ | 2.04.5± 1.1^d^ | 1.70 ± 0.8^e^ | 2.07± 1.0^f^ |
|  | LSD 0.05=104.7 |  |  |  |  |  |  |
|  | T- test | ___ | 0.57*** | -0.88*** | -1.18*** | -1.54*** | -0.71*** |
| **Superoxide dismutase**  **(U/mg)** | Mean ± SD | 614.5±18.5^a^ | 180± 17.3^b^ | 378.7± 19.4^c^ | 598.5± 19.9^d^ | 599.5± 26.5^e^ | 617±26.03^f^ |
|  | LSD 0.05=78.74 |  |  |  |  |  |  |
|  | T- test | ___ | 0.905*** | 1.267*** | 0.311*** | 0.434*** | 5.139*** |
| **Glutathione S transferase**  **GST**  **(U/mg )** | Mean ± SD | 5.8± 0.99^b^ | 2.2± 1.99^a^  59 | 3.6± 0.9^d^ | 4.3± 1.6^e^ | 4.3± 1.2^d^ | 5.02±1.9 ^f^ |
|  | LSD 0.05=1.89 |  |  |  |  |  |  |
|  | T- test | ___ | 0.716*** | -0.426*** | -2.004*** | -2.44*** | -0.204*** |
| **Lipid peroxidation**  **(mm/mg)** | Mean ± SD | 1.52±0.7^a^ | 4.8± 1.9^b^ | 2.79± 1.2^c^ | 1.70± 0.6^d^ | 1.7± 0.9^f^ | 1.5± 0.5^e^ |
|  | LSD 0.05=24.07 |  |  |  |  |  |  |
|  | T- test | ___ | -0.224 | 2.1** | 5.196** | 3.372** | 3.775*** |

Data are represented as mean ± SE. T-test values ***: significant at P<0.001. ANOVA analysis: within each row, means with different superscript (a, b, c,d or f) are significantly different at P<0.05, whereas means superscripts with the same letters mean that there is no significant difference at P>0.05. LSD: least significant difference.
