# Supplementary material for: Comparison between the Antioxidant and Antidiabetic Activity of Fenugreek and Buckthorn in Streptozotocin-Induced Diabetic Male Rats
Source: Biomed Res Int. 2021 Aug 27;2021:7202447. doi: 10.1155/2021/7202447 (PMC8420976; doi:10.1155/2021/7202447)
Supplement: Supplementary 2 — Table 2 (sup.): effect of treating streptozotocin-induced diabetes with fenugreek and buckthorn aqueous extract on glucose, glycated hemoglobin, albumin, and lactate dehydrogenase in male rats. [file 7202447.f2.docx]

Table 2 (sup): Effect of treating streptozotocin induced diabetes with fenugreek and buckthorn aqueous extract on glucose, Glycated hemoglobin, albumin and lactate dehydrogenase in male rats

| **Statistics and tests** | | **G1**  **(-ve control)** | **G2**  **(+ve control)** | **G3**  **Fenugreek leaf** | **G4**  **Fenugreek seed** | **G5**  **Buckthorn leaf** | **G6**  **Buckthorn seed** |
| --- | --- | --- | --- | --- | --- | --- | --- |
| **Glucose**  **mg/dl** | Mean ± SD | 111.5± 10.7^a^ | 231.7± 36.3^b^ | 150.3±37.7^c^ | 139.4±35.4^d^ | 134.2±40.7^e^ | 127.9±58.7^f^ |
|  | LSD 0.05=50.69 |  |  |  |  |  |  |
|  | T- test | ___ | 7.775*** | 2.427*** | 4.505*** | 1.323*** | 1.905*** |
| **Gluco-hemoglobin %** | Mean ± SD | 3.4± 0.2^b^ | 4.6± 1.3^a^ | 4.2± 1.2^c^ | 3.9± 0.1^f^ | 4.2± 1.03^d^ | 4.0± 0.96^e^ |
|  | LSD 0.05=1.36 |  |  |  |  |  |  |
|  | T- test | ___ | -2.13*** | 3.424* | 2.067*** | 3.641* | 1.615** |
| **Albumin**  **(g/dl)** | Mean ± SD | 5.9± 0.9^a^ | 3.7± 0.3^b^ | 4.5± 0.2^d^ | 5.01± 0.04^c^ | 4.7± 0.1^e^ | 5.6± 0.04^f^ |
|  | LSD 0.05=0.19 |  |  |  |  |  |  |
|  | T- test | ___ | 2.024** | -1.62 ^NS^ | -0.323* | -2.184* | -1.786** |
| **LDH**  **U/l** | Mean ± SD | 189.2± 18.3^a^ | 597.2± 20.5^b^ | 247.0± 7.6^c^ | 200.7± 16.3^d^ | 219.8± 7.7^e^ | 191.7±6.4^f^ |
|  | LSD 0.05=15.47 |  |  |  |  |  |  |
|  | T- test | ___ | 6.17*** | 1.932*** | 1.153*** | 2.408*** | 0.07*** |

Data are represented as mean ± SE. T-test values ***: significant at P<0.001. ANOVA analysis: within each row, means with different superscript (a, b, c,d or f) are significantly different at P<0.05, whereas means superscripts with the same letters mean that there is no significant difference at P>0.05. LSD: least significant difference.
